# The APSES Transcription Factor SsStuA Regulating Cell Wall Integrity Is Essential for Sclerotia Formation and Pathogenicity in *Sclerotinia sclerotiorum*

**DOI:** 10.3390/jof10040238

**Published:** 2024-03-22

**Authors:** Wenli Jiao, Maoxiang Li, Tianyi Lei, Xiaoli Liu, Junting Zhang, Jun Hu, Xianghui Zhang, Jinliang Liu, Shusen Shi, Hongyu Pan, Yanhua Zhang

**Affiliations:** 1 College of Plant Sciences, Jilin University, Changchun 130062, China; 2 Shandong Yellow River Delta National Nature Reserve Management Committee, Scientific Research Center, Dongying 257091, China; 3 College of Plant Protection, Jilin Agricultural University, Changchun 130118, China

**Keywords:** *Sclerotinia sclerotiorum*, APSES transcription factor, cell wall integrity, sclerotia, pathogenicity

## Abstract

APSES (Asm1p, Phd1p, Sok2p, Efg1p, and StuAp) family transcription factors play crucial roles in various biological processes of fungi, however, their functional characterization in phytopathogenic fungi is limited. In this study, we explored the role of SsStuA, a typical APSES transcription factor, in the regulation of cell wall integrity (CWI), sclerotia formation and pathogenicity of *Sclerotinia sclerotiorum*, which is a globally important plant pathogenic fungus. A deficiency of *SsStuA* led to abnormal phosphorylation level of SsSmk3, the key gene *SsAGM1* for UDP-GlcNAc synthesis was unable to respond to cell wall stress, and decreased tolerance to tebuconazole. In addition, Δ*SsStuA* was unable to form sclerotia but produced more compound appressoria. Nevertheless, the virulence of Δ*SsStuA* was significantly reduced due to the deficiency of the invasive hyphal growth and increased susceptibility to hydrogen peroxide. We also revealed that SsStuA could bind to the promoter of catalase family genes which regulate the expression of catalase genes. Furthermore, the level of reactive oxygen species (ROS) accumulation was found to be increased in Δ*SsStuA*. In summary, SsStuA, as a core transcription factor involved in the CWI pathway and ROS response, is required for vegetative growth, sclerotia formation, fungicide tolerance and the full virulence of *S. sclerotiorum*.

## 1. Introduction

*Sclerotinia sclerotiorum* (Lib.) de Bary, a notorious necrotrophic plant pathogenic fungus, has a complete life cycle and infection cycle with extremely wide distribution, causing blight, stem and crown rots of numerous crops [1,2,3,4]. *S. sclerotiorum* is widely distributed in warm and arid regions and can infect various plants, including oilseed crops such as soybean and rapeseed, as well as ornamental plants such as marigold and tulip [5]. Polymorphism is a remarkable characteristic of *S. sclerotiorum* which produces compound appressoria, sclerotia, apothecium and ascospore in the process of morphological differentiation [2,4,6]. Therein, compound appressorium and ascospore are an important infection structure, and sclerotia is the core component of the life and infection cycle of *S. sclerotiorum* [7].

Sclerotia are produced by *S. sclerotiorum* under unfavorable external environmental conditions, allowing them to ensure extended periods in the field. When conditions become favorable, sclerotia germinate to produce either hyphae or apothecia. Hyphae can directly infect plant leaves, while ascospores released from apothecia discs infect plant tissues, initiating a new infection cycle [8,9]. Sclerotia serve as critical structures for long-term survival and act as the primary inocula of the disease. Once the sclerotia are formed, chemical control of *S. sclerotiorum* becomes ineffective [10]. Therefore, the spread of the pathogenic fungi would be significantly diminished if sclerotia formation were to be abolished. The formation and development of sclerotia are mediated by complex internal and external factors, including pH, reactive oxygen species (ROS), and nutrient limitation. *S. sclerotiorum* relies on multiple signaling pathways, such as cAMP signaling, mitogen-activated protein (MAP) kinase pathway, and autophagy, to respond to and regulate these stimuli [11,12,13]. Furthermore, transcription factors (TFs) serve as vital mediators between signal transduction pathways and target gene expression. Fungal-specific TF families are classified into different types according to their conserved domains, including Zn2/Cys6 (Zn cluster), fungal-specific transcription factor domain, APSES, etc. [14]. Among these, APSES TFs play a pivotal role in the life process of various fungi [15].

The APSES family TFs feature a sequence-specific DNA-binding domain known as the APSES domain, which can adopt a bHLH-like structure [16]. Moreover, it has been proposed that APSES domains may be evolved from KilA-N-like precursors found in viruses that were integrated into host cells during early fungal evolution [17]. Within fungi, APSES family TFs such as Asm1p, Phd1p, Sok2p, Efg1p, and StuAp serve as pivotal regulators of various developmental processes [14,18,19]. For example, AnStuA, the first identified APSES TF, is indispensable for asexual reproduction and conidiophore development in *Aspergillus nidulans* [15]. In *Candida albicans*, APSES TFs regulate cell differentiation, morphogenesis, and metabolism [20], while Sok2 and Phd1 regulate pseudohyphal differentiation in *Saccharomyces cerevisiae* by modulating the expression of Flo11, which is crucial for filamentous growth [21]. In *A. fumigatus*, *StuA* mutants display impaired asexual reproduction and abnormal conidiophore morphology [22], and RgdA governs mycelial growth and virulence [23]. Similarly, *GcStuA*, *StuA* homolog in *Glomerella cingulata*, is involved in maintaining appressorium turgor pressure and virulence [24]. The *StuA* homolog *Mstu1* in *Magnaporthe oryzae* is essential for pathogenicity [25], while deletion of *FgStuA* in *Fusarium graminearum* results in reduced spore production, pathogenicity, and secondary metabolite production [26]. MoSwi6 interacts with MoMps1 and is necessary for mycelial growth, conidial morphogenesis, and pathogenicity in *M. oryzae* [27].

In conclusion, the APSES protein family plays crucial roles in biological processes, such as sporulation, cell differentiation, hyphal growth, secondary metabolism, and virulence [15]. However, the regulatory mechanism of APSES TFs on the growth and development of *S. sclerotiorum* remains unclear. This study focused on characterizing SsStuA as an APSES family TF, which serves as a pivotal transcription factor regulating normal growth, sclerotia formation, and full virulence in *S. sclerotiorum*. Studying the function of SsStuA aids in further understanding of the underlying molecular mechanisms that regulate the growth and pathogenicity of *S. sclerotiorum*. This research also offers valuable insights for the prevention and treatment of the diseases caused by this destructive phytopathogen.

## 2. Materials and Methods

### 2.1. Culture Condition of Strains

*Sclerotinia sclerotiorum* UF-1 was used as the wild type (WT) strain [28]. WT, Δ*SsStuA* and Δ*SsStuA*-C strains were routinely grown on potato dextrose agar (PDA) (potato 200 g, glucose 20 g, agar 17 g, constant volume to 1 L) in the dark, at 25 °C constant temperature incubator.

### 2.2. Identification and Sequence Analysis of SsStuA

Phylogenetic analysis of SsStuA was performed by MEGA version 7.0 software using the neighbor-Joining method with 1000 bootstrap replicates. The conserved domains of SsStuA (XP_001590416.1) and its homologs (*Botrytis cinerea* B05.10/XP_024548075.1, *Colletotrichum siamense*/KAF4880364.1, *Magnaporthe oryzae* 70-15/XP_003718315.1, *Aspergillus nidulans* FGSC A4/XP_050467081.1, *Aspergillus fumigatus* Af293/XP_755125.1, *Cercospora beticola*/XP_023454930.1, *Fusarium graminearum* PH-1/XP_011319067.1, *Ustilago maydis*/XP_011388143.1) were analyzed by NCBI (https://www.ncbi.nlm.nih.gov/, accessed on 10 March 2023) and Interpro (http://www.ebi.ac.uk/interpro, accessed on 10 March 2023), and the distribution of the conserved domain was visualized by GPS version 2.0 software [29]. The amino acid sequences of the conserved domain in SsStuA and other fungal species underwent alignment using Clustal W version 1.7 [30].

### 2.3. SsStuA Gene Knockout and Complementation

The knockout strategy for the *SsStuA* gene is presented in Figure S1A. Knockout fragments were generated using the SPLIT-MARKER PCR method: the upstream (FR1) and downstream fragments (FR2) of SsStuA were amplified using primers SsStuA F1/R1 and SsStuA F2/R2, respectively. Fragments HY and YG were obtained from the pUCATPH vector using primers M13R/NLC37 and M13F/NLC38 [31,32]. Overlapping fragments were fused using primers SsStuA F1/NLC37 and NLC38/SsStuA R2. These two fragments were then transformed in protoplasts of UF-1 of *S. sclerotiorum*, as described previously [7,33], and transformants’ hyphal tips were selected using hygromycin (100 μg/mL) at least five times.

Complementary fragments were amplified using primers SsStuA CF1/CR1 and SsStuA CF2/CR2. The two fragments were co-transformed into protoplasts of Δ*SsStuA* mutants together with the geneticin fragment amplified using primers DW69/DW70 from the pII9 vector. The full SsStuA sequence was amplified from wild-type (WT) genomic DNA with the upstream and downstream regions. Hyphal tips of transformants were selected using geneticin (200 μg/mL). Δ*SsStuA* and Δ*SsStuA*-C were confirmed via PCR and quantitative real-time PCR. Details of all primers used in this study are presented in Table S1.

### 2.4. Plasmid Constructs and Transformation

The vector *pNAH-ONG* containing a GFP tag was used to see the protein expression in the cell. The SsStuA cDNA sequence was amplified using ONG-SsStuA F/R primers and incorporated into the *pNAH-ONG* vector, which also contains the neomycin phosphotransferase gene, using the ClonExpress II One Step Cloning Kit (Vazyme Biotech Co., Ltd., Nanjing, China). Subsequently, the constructed vector was transformed into the protoplasts of stains UF-1, and transformants’ hyphal tips were purified through multiple rounds of selection (at least five times) with geneticin (200 μg/mL).

### 2.5. Stress Treatment

Cell wall synthesis inhibitors including Congo Red (CR, 500 μg/mL), sodium dodecyl sulfate (SDS, 0.01%) and Calcofluor White (CFW, 50 μg/mL) were added to PDA to explore whether deficiency of *SsStuA* affects cell wall formation in *S. sclerotiorum*. CM medium was composed of 0.1 g KH_2_PO_4_, 0.125 g MgSO_4_·7H_2_O, 0.075 g NaCl, 0.5 g Ca(NO_3_)_2_·4H_2_O, 5 g glucose, 0.5 g yeast extract, 0.5 g casein hydrolysate, and 10 g agar per 500 mL. MM medium, a modification of CM, lacked 0.5 g yeast extract and 0.5 g casein hydrolysate. Additionally, MM-N medium was formulated by excluding Ca(NO_3_)_2_·4H_2_O from MM, while MM-C medium omitted glucose. Furthermore, MM-P medium was prepared by eliminating KH_2_PO_4_ from MM. These various nutrient-deficient media were used in the study. Agar disks (d = 0.5 cm) of wild-type, Δ*SsStuA* and Δ*SsStuA*-C strains were inoculated and the culture dishes were cultivated in the dark, at 25 °C constant temperature incubator, the mycelial growth was measured after 2d inoculation and the inhibition rate was calculated as: inhibition rate = (diameter of untreated − diameter of stress treated)/diameter of untreated × 100%.

### 2.6. Evaluation of ROS and Oxalic Acid Accumulation

To analyze the effects of oxidative stress, wild-type, Δ*SsStuA* and Δ*SsStuA*-C strains were inoculated on PDA medium supplemented with different concentrations of H_2_O_2_ (0, 5 and 15 mM). Mycelial growth diameters were measured after 2d inoculation at each concentration of H_2_O_2_ repeated three times. 3,3′-Diaminobenzidine Tetrahydrochloride solution (DAB) and nitroblue tetrazolium (NBT) staining solution were used for qualitative analysis of H_2_O_2_ and O_2_^−^ accumulation. Mycelial agar disks (d = 0.5 cm) of wild-type, Δ*SsStuA* and Δ*SsStuA*-C strains were placed in a 24-well plate, 1mL DAB (1 mg/mL) was added, and the staining was compared with that after 2d inoculation, then photographed. The mycelia of WT, Δ*SsStuA* and Δ*SsStuA*-C strains were stained with NBT (0.5 mg/mL) for 20 min, and the staining was compared and photographed. 100 μg/mL bromophenol blue was added to PDA to detect oxalic acid production and photographed after 2 days of inoculation. If the color of the medium has changed from blue to yellow, it indicates that the strain can produce oxalic acid normally. 

### 2.7. Analysis of Sclerotia and Compound Appressoria Formation of S. sclerotiorum

Compound appressoria of WT, Δ*SsStuA* and Δ*SsStuA*-C were observed on glass slides by inoculating agar disks (d = 0.7 cm) and cultivated in a humidifier box at 25 °C incubator. After 48 h incubation, taken pictures and calculated the area of compound appressoria using Image J version 1.50i [29]. The morphology of compound appressoria of WT, Δ*SsStuA* and Δ*SsStuA*-C strains on glass slides was observed by microscopy. Onion epidermis was used to observe the penetration efficiency difference between the compound appressoria of wild-type, Δ*SsStuA* and Δ*SsStuA*-C strains. The onion epidermis was inoculated with agar disks (d = 0.5 cm) of strains and cultivated in a humidifier box, after 12 h and 24 h incubation, the invasion hypha was stained with lactophenol cotton blue (Sigma, St. Louis, MA, USA) for 2 min, then rinsed with distilled water and observed by DIC microscopy. The morphology, number, and weight of sclerotia were photographed and calculated after 2 weeks incubation. 

### 2.8. Pathogenicity Analysis

Pathogenicity analysis of wild-type, Δ*SsStuA* and Δ*SsStuA*-C were analyzed on different hosts. Mycelial agar disks (d = 0.5 cm) were inoculated on the leaves and cultivated in a humidifier box at 25 °C constant temperature incubator. After 48 h inoculation, the areas of lesions on wounded and unwounded leaves were calculated by Image J version 1.50i [29].

### 2.9. Quantitative Real-Time PCR Analysis

The total RNA of strains was extracted by TransZol Up Plus RNA Kit (TransGen Biotech, Beijing, China). The relative expression of genes was measured by Green qPCR SuperMix (TransGen Biotech, Beijing, China). All the primers used in this article are shown in Table S1 and the name was remarked with “Q”. The qRT-PCR was performed by PrimePro^48^ (Serial NO. R000100174).

### 2.10. Protein Extraction and Western Blot

Mycelia of wild-type and Δ*SsStuA* were cultured in PD for 36 h and supplemented with CR (500 μg/mL) as cell wall stress for 2 h, then collected and ground, protein extraction solution (10 mM Tris-HCl, 150 mM NaCl, 0.5 mM EDTA, 1% Triton X-100, 1 mM PMSF, pH 7.5) 1 mL containing 10 μL protease inhibitor and 10 μL phosphatase inhibitor. The lysate inserted in the ice for 20 min and centrifuged at 12,000 rpm for 15 min at 4 °C. The supernate with equal volumes of 2× loading buffer was separated by 12% sodium dodecyl sulfate–polyacrylamide gel electrophoresis (SDS-PAGE) and transferred to a polyvinylidene fluoride membrane. The Phospho-p44/42 MAPK antibody (Cell Signaling Technology, Boston, MA, USA) was used at a 1:3000 dilution for detecting the phosphorylated SsSmk1 and SsSmk3. The second antibody was HRP-labeled goat antirabbit IgG (1:5000) and Coomassie Brilliant Blue (CBB) was used as a control.

### 2.11. Yeast One-Hybrid

The cDNA sequence of SsStuA was amplified by AD-SsStuAF/AD-SsStuAR and cloned into pGADT7 though ClonExpress II One Step Cloning Kit. The promoters of *Sscle_01g011570*, *Sscle_04g037170*, *Sscle_15g107280*, *Sscle_05g044180*, *Sscle_08g064900*, *Sscle_15g104430*, *Sscle_05g047950* and *Sscle_03g026200* were amplified and cloned into pHIS. pGADT7-SsStuA and pHIS-X were transformed into yeast Y187 strain. 3-amino-1,2,4-triazole (3-AT, 30 mM) was used to inhibit self-activation of pHIS-X.

## 3. Results

### 3.1. SsStuA Is a Critical APSES TF Regulating Sclerotia Formation in S. sclerotiorum 

**Figure 1:**
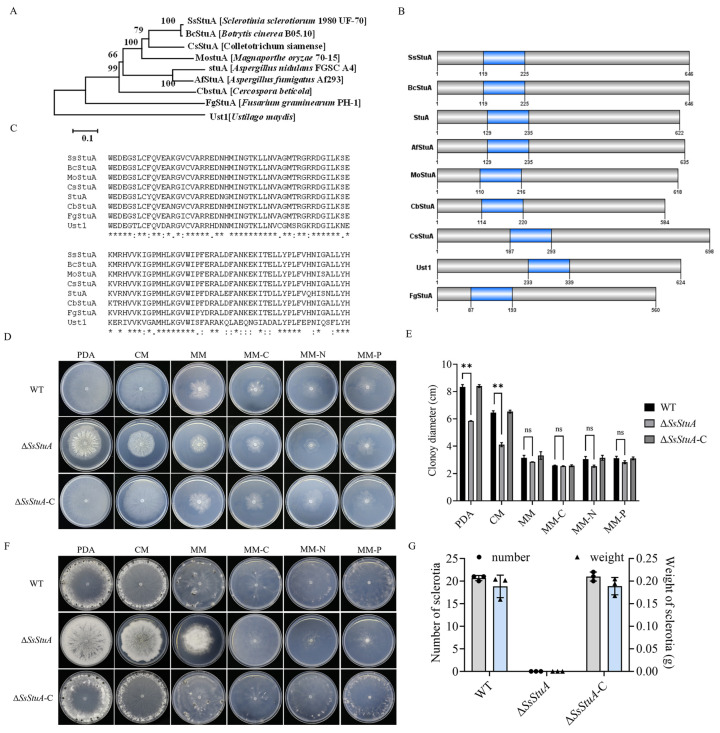
*SsStuA* performed a critical role in vegetative growth and sclerotia formation of *S. sclerotiorum*. (**A**) Phylogenetic analysis of SsStuA. The sequences of SsStuA and its homologs were aligned by ClustalW, and MEGA 7 was used to construct the phylogenetic tree by using the neighbor-Joining method with 1000 bootstrap replicates. (**B**) Analysis of conserved domains of SsStuA. Conserved domains were analyzed by interpro and visualized by GPS 2.0. (**C**) The conserved sites in the domain of SsStuA. The conserved sites were labeled with an asterisk “*”. (**D**) Colony morphology of Δ*SsStuA*, WT and Δ*SsStuA*-C. (**E**) Colony diameters were measured after 48 h incubation. (**F**,**G**) Sclerotia formation of Δ*SsStuA*, WT and Δ*SsStuA*-C (Gray and blue represent the number and weight of sclerotia in (**G**), respectively). Error bars mean standard deviations (SDs) and significant differences between the WT and Δ*SsStuA* were performed by *t* test (**, *p* < 0.01), ns means that there was no significance between WT and Δ*SsStuA*.

SsStuA (Sscle_10g075100) was grouped together with the *Botrytis cinerea* BcStuA sequence, which revealed 97.83% similarity (Figure 1A). SsStuA and its homologous proteins from other fungal species contained a conserved domain named KilA-N domain (pfam04383) (Figure 1B,C). In order to clarify the biological function of *SsStuA* in *S. sclerotiorum*, split-marker PCR was used to delete *SsStuA*, and Δ*SsStuA* was then complemented by transformation with a copy of *SsStuA* (Figure S1). There were obvious differences in the morphology between UF-1 and Δ*SsStuA* on PDA (Figure 1D), and the radial growth of Δ*SsStuA* was reduced (Figure 1E). Significantly, only deep and dense colonies of Δ*SsStuA* were observed after 14 days of culture on PDA, CM, and MM, indicating that the deficiency of SsStuA abolishes the formation of sclerotia (Figure 1F,G). The results indicate that *SsStuA* performs a vital function in the vegetative growth and sclerotia formation of *S. sclerotiorum*.

### 3.2. SsStuA Is Involved in Cell Wall Integrity of S. sclerotiorum

**Figure 2:**
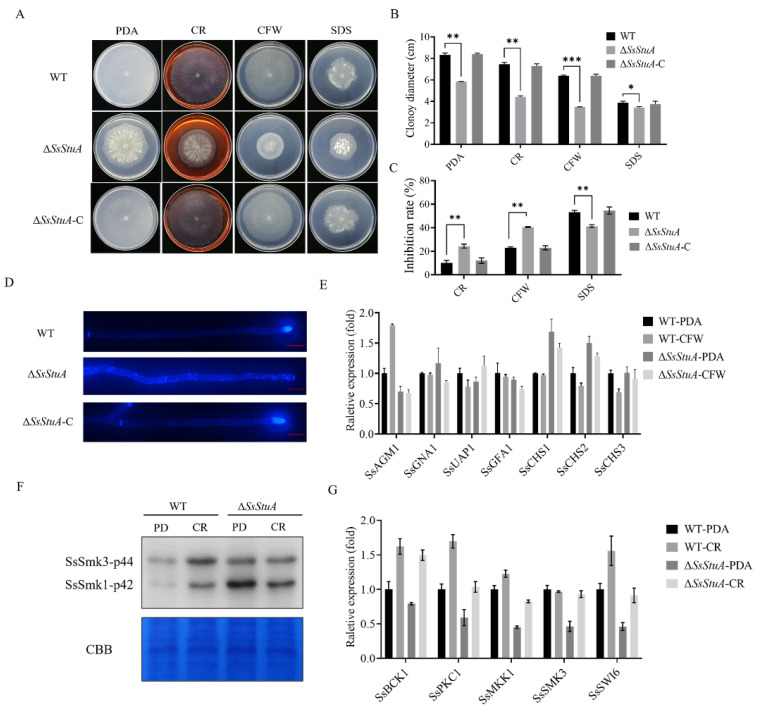
SsStuA contributes to cell integrity of *S. sclerotiorum*. (**A**) Colony morphology of Δ*SsStuA*, WT and Δ*SsStuA*-C under different cell wall inhibitors. (**B**) Colony diameters of Δ*SsStuA*, WT and Δ*SsStuA*-C. (**C**) The inhibition rate of hyphal growth under diverse cell wall inhibitors. Inhibition rate = (diameter of untreated − diameter of stress treated)/(diameter of untreated × 100%). (**D**) Deficiency of *SsStuA* caused defective cell wall. Δ*SsStuA*, WT and Δ*SsStuA*-C were stained with Calcofluor White (CFW) and observed by fluorescence microscope. Scale bar = 50 μm. (**E**) Relative expression of genes involved in UDP-GlcNAc synthesis pathway and chitin synthesis. (**F**) Phosphorylation level of SsSmk3 in WT and Δ*SsStuA* under CR treatment. (**G**) Relative expression of CWI pathway genes in WT and Δ*SsStuA* under CR treatment. Error bars represent the SDs and significant differences between the WT and Δ*SsStuA* were performed by *t* test (*, *p* < 0.05; **, *p* < 0.01, ***, *p* < 0.001).

Since Δ*SsStuA* showed a compact colony on PDA, we suspected that SsStuA might affect the cell wall integrity. The CWI (cell wall integrity) signaling pathway provides a pivotal role in responding to diverse stress in pathogenic fungi [34,35]. Cell wall inhibitors (CR and CFW) limited the growth of Δ*SsStuA* (Figure 2A–C) and CFW staining hyphae exhibited scattered and distributed blue fluorescence (Figure 2D), which suggest that *SsStuA* is involved in cell wall integrity of *S. sclerotiorum*. UDP-GlcNAc is the precursor of chitin which is the main component of the fungal cell wall. Therefore, we assessed the expression level of *SsAGM1* under CFW treatment. *SsAGM1* responded to CFW treatment and was up-regulated in WT, but there was no significant change in Δ*SsStuA* (Figure 2E). These results suggest that the chitin synthesis process in Δ*SsStuA* is affected. The genes associated with chitin synthesis (*SsCHS1* and *SsCHS2*) exhibited significantly elevated expression levels in Δ*SsStuA* (Figure 2E), potentially accounting for the dense colony phenotype in Δ*SsStuA*.

Moreover, the cell wall integrity pathway is regulated by MAPK-SsSmk3. After treatment with CR, the phosphorylation level of SsSmk3 significantly increased in wild type, but there was no significant change in Δ*SsStuA* (Figure 2F). The expression levels of CWI pathway related genes (*SsPKC1*, *SsMKK1*, *SsSMK3* and *SsSWI6*) in Δ*SsStuA* were decreased remarkably compared with that of WT (Figure 2G). In summary, transcription factor SsStuA is involved in the CWI pathway.

### 3.3. ΔSsStuA Is Sensitive to Tebuconazole 

**Figure 3:**
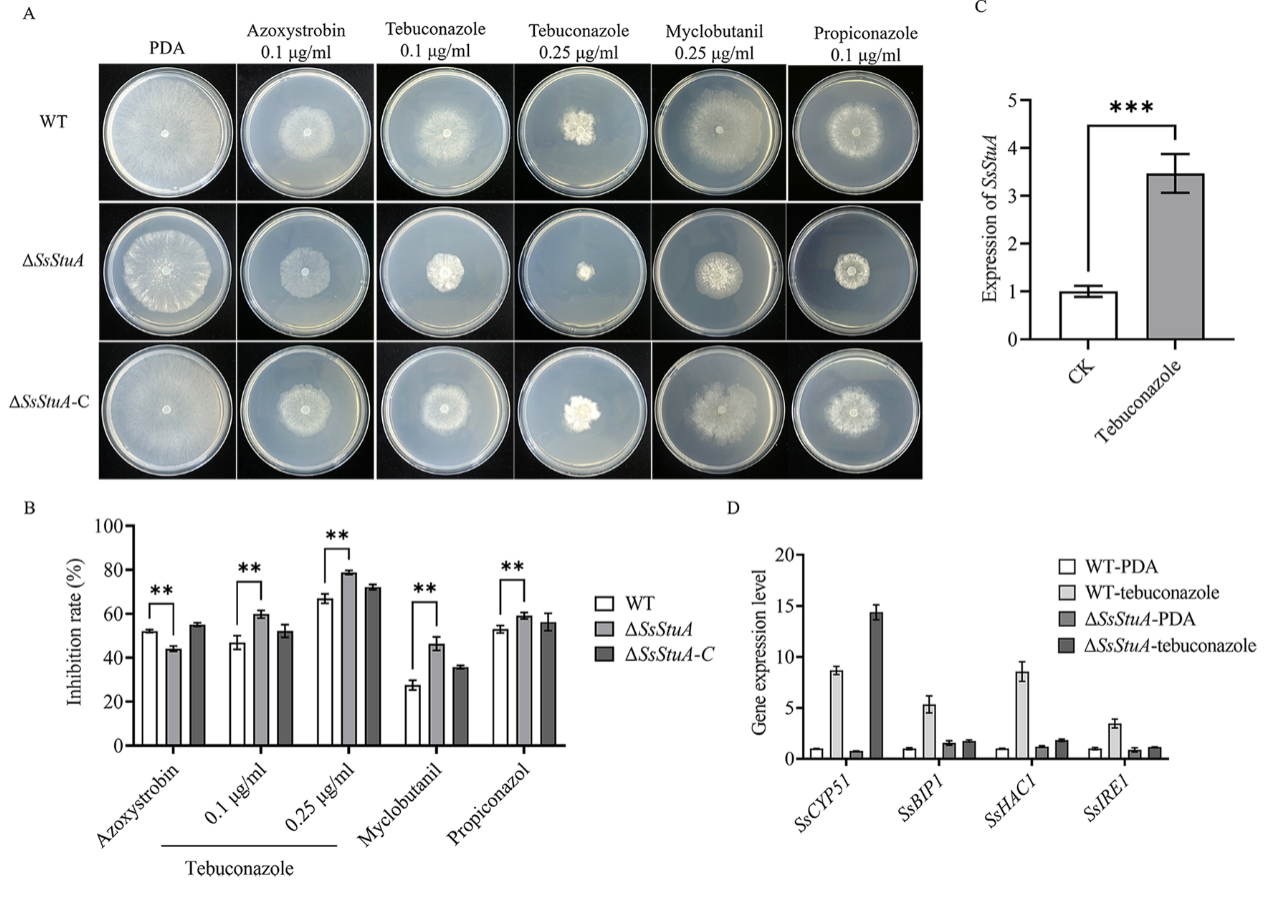
Δ*SsStuA* increased sensitivity to tebuconazole. (**A**) Sensitivity of Δ*SsStuA* to fungicides. (**B**) Inhibition rate of fungicides on the Δ*SsStuA*. Inhibition rate = (diameter of untreated − diameter of fungicide treated)/(diameter of untreated × 100%). (**C**) The expression of *SsStuA* under tebuconazole treatment. (**D**) Gene expression of WT and Δ*SsStuA* under tebuconazole treatment. Error bars represent the SDs and significant differences were performed by *t* test (**, *p* < 0.01, ***, *p* < 0.001).

Tebuconazole is a triazole fungicide belonging to DMI class, which is an ergosterol inhibitor of phytopathogens that mainly affects the formation of fungal cell wall [36,37]. The deletion of *SsStuA* caused defects in the CWI pathway, which led to the question of whether Δ*SsStuA* was sensitive to fungicides. Thus, different fungicides were added to explore whether the *SsStuA* affects the fungicide resistance of *S. sclerotiorum.* The inhibition rate of tebuconazole, myclobutanil and propiconazole was all increased in Δ*SsStuA* (Figure 3A,B), and the expression level of *SsStuA* also increased under tebuconazole treatment (Figure 3C). *CYP51* is the target of tebuconazole [38,39], therefore, the expression of *SsCYP51* was measured in WT and Δ*SsStuA*. The results showed that *SsCYP51* was up-regulated in Δ*SsStuA*, which indicate that *SsCYP51* is not the only target of tebuconazole in *S. sclerotiorum* (Figure 3D). Tebuconazole triggers endoplasmic reticulum (ER) stress by activating the unfolded protein response (UPR) [40]. To determine whether *SsStuA* is important for the UPR, the expression levels of *SsBIP1*, *SsHAC1* and *SsIRE1* were measured. Compared to WT, the deficiency of *SsStuA* caused no obvious change of *SsBIP1*, *SsHAC1* and *SsIRE1* under tebuconazole treatment (Figure 3D), demonstrating that *SsStuA* is essential for the UPR.

### 3.4. ΔSsStuA Mutant Shows Attenuation of Pathogenicity 

**Figure 4:**
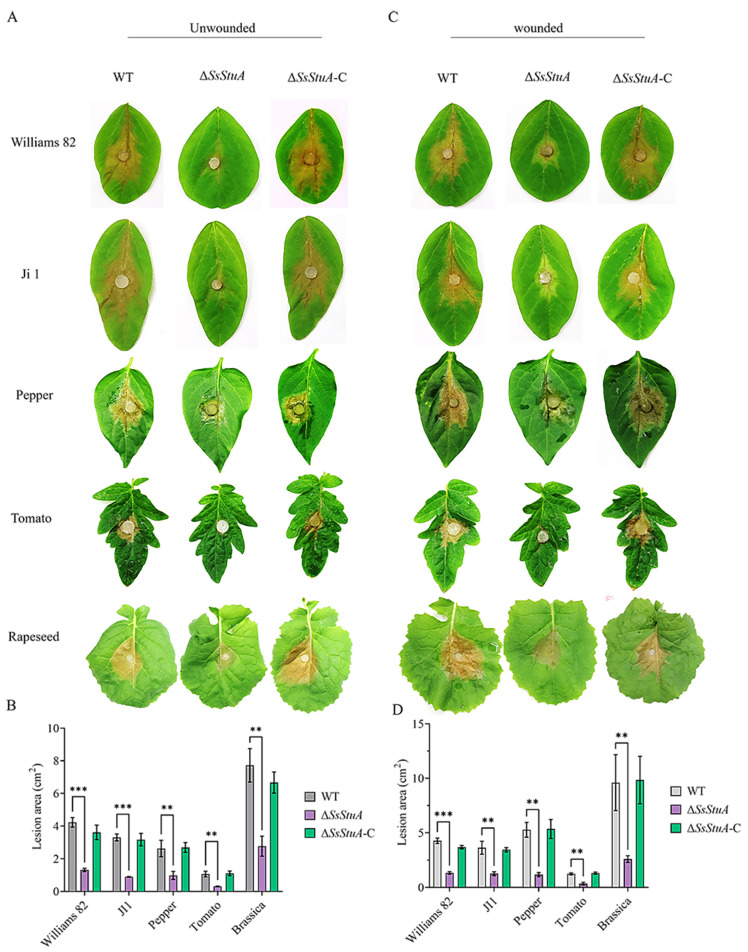
*SsStuA* influenced the full virulence of *S. sclerotiorum*. (**A**) Unwounded inoculation of Δ*SsStuA*, WT, and Δ*SsStuA*-C on different host plants. (**B**) Analysis of the lesion area under unwounded inoculation. (**C**) Wounded inoculation of Δ*SsStuA*, WT, and Δ*SsStuA*-C on different host plants. (**D**) Analysis of the lesion area under wounded inoculation. The lesion area was calculated by Image J. Error bars represent the SDs and significant differences between the WT and Δ*SsStuA* were performed by *t* test (**, *p* < 0.01, ***, *p* < 0.001).

**Figure 5:**
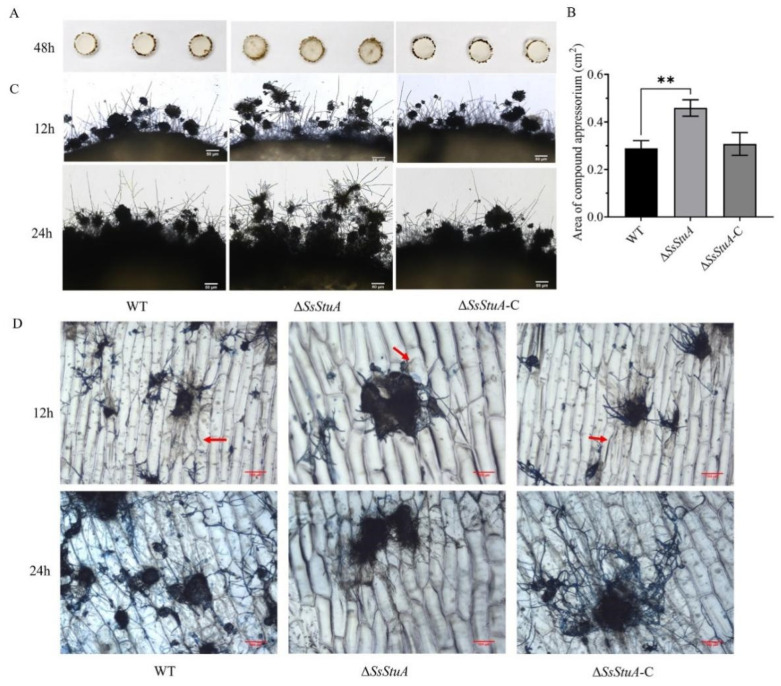
Compound appressoria were abnormal in Δ*SsStuA*. (**A**) Compound appressoria production of strains on glass slides. Photographed at 2 days post-inoculation. (**B**) The area of compound appressoria produced by Δ*SsStuA* was larger than that of WT and Δ*SsStuA*-C. Area of compound appressoria was calculated by Image J. Error bars represent the SDs and significant difference was performed by *t* test (**, *p* < 0.01). (**C**) Morphology of compound appressoria on glass slides. Scale bar = 50 μm. (**D**) The invasive hyphae of Δ*SsStuA* showed retarded growth in onion epidermis. The compound appressoria were stained in blue by lactophenol blue, the red arrow points to the invasive hyphae. Scale bar = 100 μm.

The pathogenicity of Δ*SsStuA* was obviously decreased on unwounded leaves (Figure 4A,B), Δ*SsStuA* inoculated on wounded leaves also caused defects in pathogenicity (Figure 4C,D). Compound appressorium is a prerequisite for successful penetration of the host organism by *S. sclerotiorum*. Therefore, the formation and development of compound appressoria were investigated on glass slide, and obviously numerous compound appressoria were formed from the agar disk of Δ*SsStuA* (Figure 5A,B). We further observed the morphology of compound appressoria, but there was no distinction between Δ*SsStuA* and WT (Figure 5C). In order to compare the penetration capacity of compound appressoria, WT, Δ*SsStuA* and Δ*SsStuA*-C were inoculated on onion epidermis respectively and stained with lactophenol cotton blue to observe the growth situation of the invasive hyphae (IH). The unstained IH of Δ*SsStuA* showed retarded growth than that of WT and Δ*SsStuA*-C (Figure 5D). The results indicate that *SsStuA* probably negatively regulates the formation of compound appressoria but positively regulates the growth of IH in plant tissue, and the deletion of *SsStuA* perhaps affects the effects of other pathogenic factors besides compound appressoria.

### 3.5. ΔSsStuA Was Hypersusceptible to Hydrogen Peroxide

**Figure 6:**
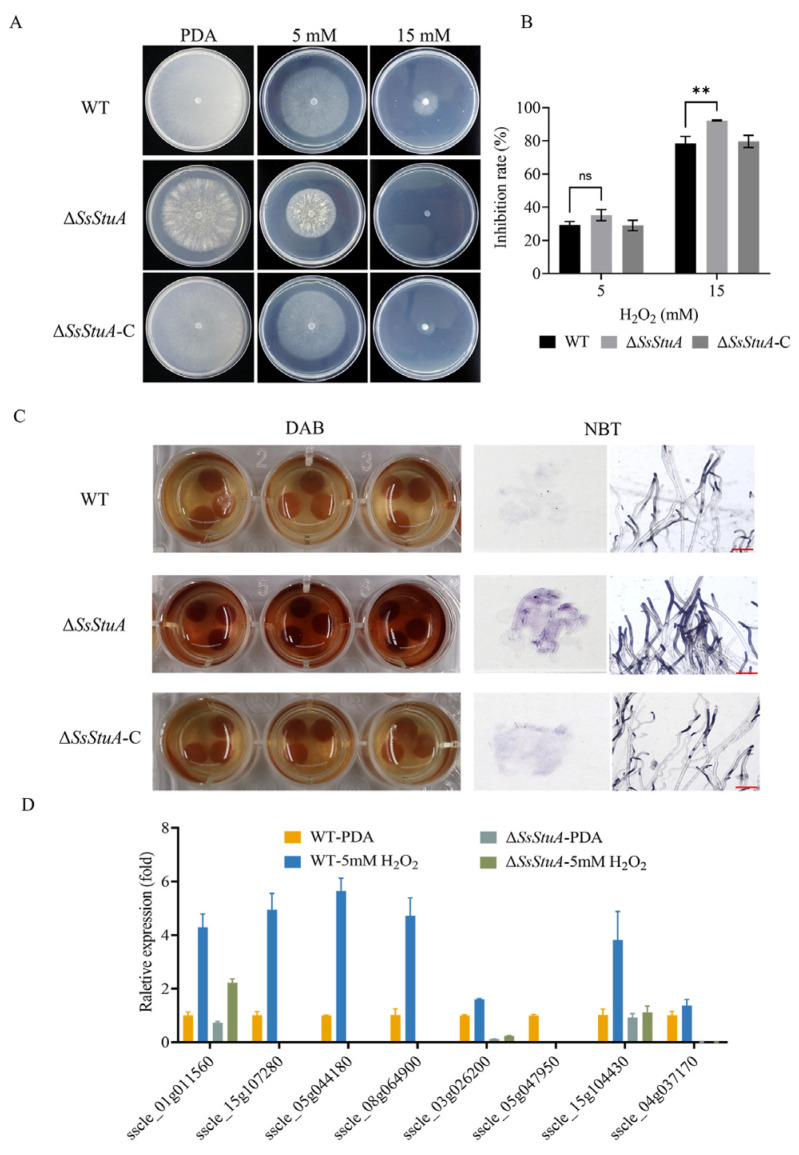
Δ*SsStuA* was hypersusceptible to H_2_O_2_. (**A**) The morphological phenotype of WT, Δ*SsStuA* and Δ*SsStuA*-C in PDA medium supplemented with 0, 5 and 15 mM H_2_O_2_ concentrations. (**B**) The hyphal growth of WT, Δ*SsStuA* and Δ*SsStuA*-C at 0, 5 and 15 mM H_2_O_2_ concentrations. Colony diameters were measured at 2 days post-inoculation. (**C**) Detection of ROS accumulation in hyphae of strains. DAB and NBT were used to detect ROS accumulation. Scale bar = 50 μm. (**D**) The relative gene expression of CAT family genes in WT and Δ*SsStuA*. Error bars represent the SDs and significant difference was performed by *t* test (**, *p* < 0.01), ns means that there was no significant difference between WT and Δ*SsStuA*.

The development of invasive hyphae in host tissue could respond to complex signals. Hypersensitive reaction is one of the earliest responses of plants to pathogens, which could accumulate a lot of reactive oxygen species [41]. To determine the mechanism of the reduced virulence of Δ*SsStuA*, we examined the susceptibility of Δ*SsStuA* to hydrogen peroxide. Δ*SsStuA* was more hypersusceptible to hydrogen peroxide than that of WT and Δ*SsStuA*-C strains (Figure 6A,B). Subsequently, we used DAB and NBT staining to detect ROS production during hyphal growth in Δ*SsStuA*. Compared with WT strain, the dark-brown coloration and dark-blue formazan precipitates of Δ*SsStuA* suggest an increased accumulation of ROS in Δ*SsStuA* under the same growth condition (Figure 6C). Moreover, the expression of catalase family genes was dramatically decreased in Δ*SsStuA* (Figure 6D), indicating that SsStuA is required for the normal expression of genes associated with reactive oxygen scavenging enzymes.

### 3.6. SsStuA Transcriptional Activation of Catalase Gene Expression

**Figure 7:**
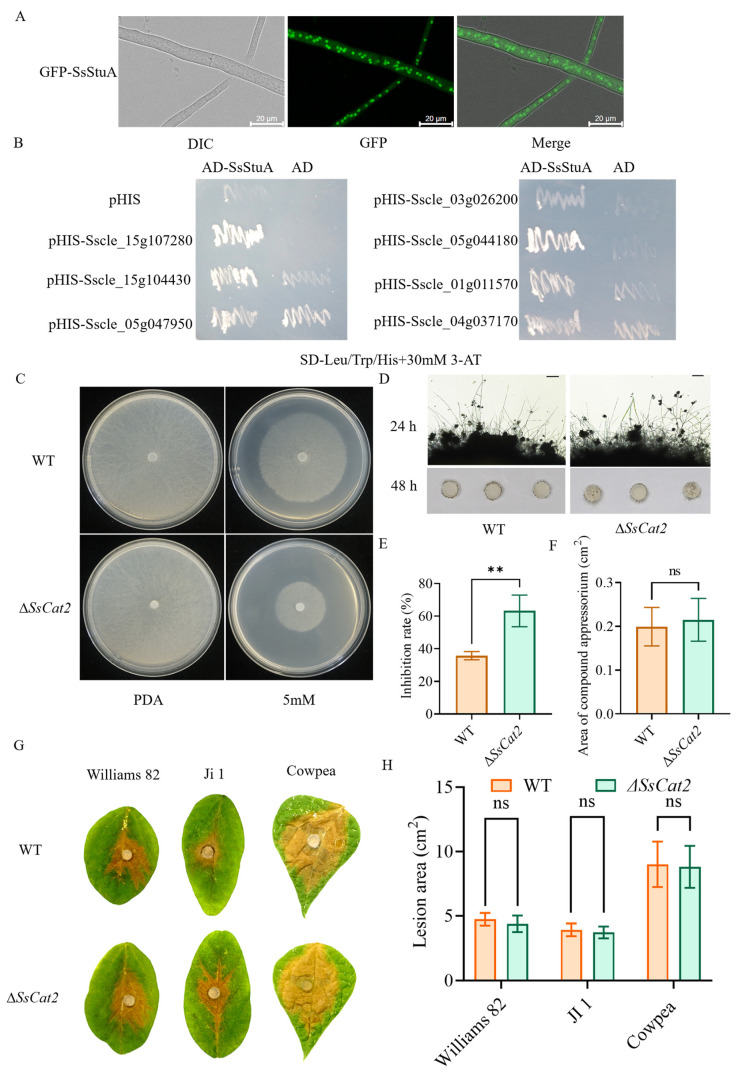
SsStuA transcriptionally activates catalase genes expression. (**A**) GFP-SsStuA was localized in the nucleus of *S. sclerotiorum*. (**B**) SsStuA binds to the promoters of the CAT family genes. (**C**,**E**) Δ*SsCat2* was hypersusceptible to H_2_O_2_. (**D**,**F**) The compound appressoria of Δ*SsCat2* did not differ from the wild type. Scale bar = 50 μm. (**G**,**H**) Pathogenicity between WT and Δ*SsCat2* does not have any obvious differences. Error bars represent the SDs and significant difference was performed by *t* test (**, *p* < 0.01), ns means that there was no significant difference between WT and Δ*SsCat2*.

SsStuA was observed to be localized in the nucleus (Figure 7A). Furthermore, there was a notable increase in the accumulation of reactive oxygen species (ROS) in Δ*SsStuA*, coupled with a down-regulation of ROS metabolism genes. A yeast one-hybrid (Y1H) experiment was performed to elucidate the reason behind the significant down-regulation of catalase (CAT) family genes in Δ*SsStuA*. The yeast strains harboring pGADT7-SsStuA and pHIS-X (*Sscle_01g011570*, *Sscle_04g037170*, *Sscle_15g107280*, *Sscle_05g044180*, *Sscle_15g104430*, *Sscle_05g047950*, and *Sscle_03g026200*) exhibited normal growth on SD-Leu/Trp/His medium supplemented with 30 mM 3-AT, while the strains containing only pGADT7 and pHIS-X failed to survive (Figure 7B). This Y1H result preliminarily confirms that SsStuA possesses transcriptional activation activity and can bind to the promoters of CAT family genes. In order to determine whether CAT genes participated in the active oxygen metabolism of *S. sclerotiorum*, the *SsCat2* gene was knocked out. Further analysis of hydrogen peroxide sensitivity and pathogenicity showed that after *SsCat2* deletion, the sensitivity to hydrogen peroxide increased (Figure 7C,E), however, the compound appressoria and pathogenicity of Δ*SsCat2* had no obvious difference with WT (Figure 7D,F–H). The results indicate that the transcription of CAT genes is regulated by SsStuA and the function of CAT family genes is redundant.

## 4. Discussion

Transcription factors (TFs) play a crucial role in signal transduction, serving as the vital link between signal reception and the expression of target genes. They regulate gene expression within cells, ultimately influencing cell function [14]. StuA belongs to the APSES protein family, which is unique to fungi and has been identified as a key regulator of cellular development and other biological processes [15,16,42]. Here, we found that SsStuA as a core transcription factor, plays vital roles in regulating CWI pathway, ROS response, tolerance to tebuconazole, sclerotia formation and the full virulence of *S. sclerotiorum*.

Sclerotia, as the initial infection source of stem rot produced by *S. sclerotiorum*, play an important role during the disease cycle [4,8]. We reported that the formation of sclerotia was abolished, and the colony showed a deeper color with increasing culture time after *SsStuA* deletion. Comparably, in *Verticillium dahliae*, Vst1 is involved in the production of microsclerotia [42]. In *A. nidulans*, StuA was required for correct differentiation and spatial organization of cell pattern in the complex conidiophore [43,44], and *MrStuA* regulated sporulation as well as vegetative growth and virulence in *Metarhizium robertsii* [45]. In the current study, we demonstrated that SsStuA plays an indispensable role in the asexual structure formation of *S. sclerotiorum*, indicating its function is conserved in ascomycetes.

Compound appressoria and Oxalic acid (OA) are the important factors that affect the full virulence of *S. sclerotiorum* [10,46,47]. Interestingly, the mechanism of sclerotia development is obviously opposite to that of compound appressoria in Δ*SsStuA*. Δ*SsStuA* produced more compound appressoria than the wild type and generated oxalic acid normally (Figure S2). However, the pathogenicity of Δ*SsStuA* was obviously decreased. Analyzing the penetrability of the compound appressorium between Δ*SsStuA* and WT, the IH of Δ*SsStuA* were grew slowly, which made the lesion area of the *SsStuA* mutant significantly lower than that of the wild type and Δ*SsStuA*-C. These results indicate that compound appressorium formation does not always dominate the pathogenicity. Furthermore, StuA had some distinctions between different ascomycetes. Mstu1 did not affect the number of appressoria but was deficient in the appressorium of *Magnaporthe grisea* [25]. Vst1 is dispensable for pathogenicity in *V. dahliae* and *V. nonalfalfae* [42]. Additionally, the pathogenicity of phytopathogenic fungus is also related to the external environment. Plants produce reactive oxygen species after inoculation with pathogens [48,49]. Δ*SsStuA* was more sensitive to reactive oxygen species than wild type and Δ*SsStuA*-C. The results further indicate that SsStuA could bind to the promoters of the CAT family genes and regulate the expression level of the CAT family genes. However, deficiency of *SsCAT2* gene does not influence the full virulence of *S. sclerotiorum*, further suggesting that the influence of *SsStuA* on pathogenicity is multifactorial.

The prevention and control of sclerotinia disease caused by *S. sclerotiorum* was still challenging. Results showed that Δ*SsStuA* was sensitive to tebuconazole, which is an ergosterol inhibitor that affects the formation of fungal cell wall by inhibiting the demethylation reaction of ergosterol intermediates. Cell wall integrity provides a pivotal role in the response and adaptation to various stresses. *SsAGM1*, the main gene that regulates uridine diphosphate-*N*-acetylglucosamine synthesis, was down-regulated in Δ*SsStuA*. Additionally, the chitin distributed randomly in the hyphae of Δ*SsStuA*, suggesting that *SsStuA* is involved in the CWI pathway. *CYP51* has been reported as the target of tebuconazole, however, *SsCYP51* was up-regulated in Δ*SsStuA* which suggests that there is a new target of tebuconazole in S. sclerotiorum, and SsStuA, a fungus-specific TF, can be developed as a new drug target for disease control.

In summary, we identified the APSES transcription factor SsStuA, as a key TF, affecting cell wall integrity, tolerance to fungicide and ROS metabolism, thereby regulating mycelial growth, sclerotium production and full virulence of *S. sclerotiorum*.

## Data Availability

Data are contained within the article and Supplementary Materials.

## Supplementary Materials

The following supporting information can be downloaded at: https://www.mdpi.com/article/10.3390/jof10040238/s1, Figure S1. Identification of mutants by PCR and qPCR. (A) The knockout strategy of *SsStuA* gene. (B) Knockout of *SsStuA* was verified by PCR. (C) The knockout and complementation mutants of *SsStuA* were verified by PCR. Error bars represent the SDs and significant difference was performed by *t* test (***, *p* < 0.001). Figure S2. Δ*SsStuA* produced oxalic acid normally. Photographs were taken at 2 days post-inoculation. Table S1. Primers used in this paper.
