# Supplementary material for: The APSES Transcription Factor SsStuA Regulating Cell Wall Integrity Is Essential for Sclerotia Formation and Pathogenicity in Sclerotinia sclerotiorum
Source: J Fungi (Basel). 2024 Mar 22;10(4):238. doi: 10.3390/jof10040238 (PMC11051248; doi:10.3390/jof10040238)
Supplement: Supplementary file 1 [file jof-10-00238-s001.zip › jof-2902875-supplementary.pdf]

Supplementary Materials

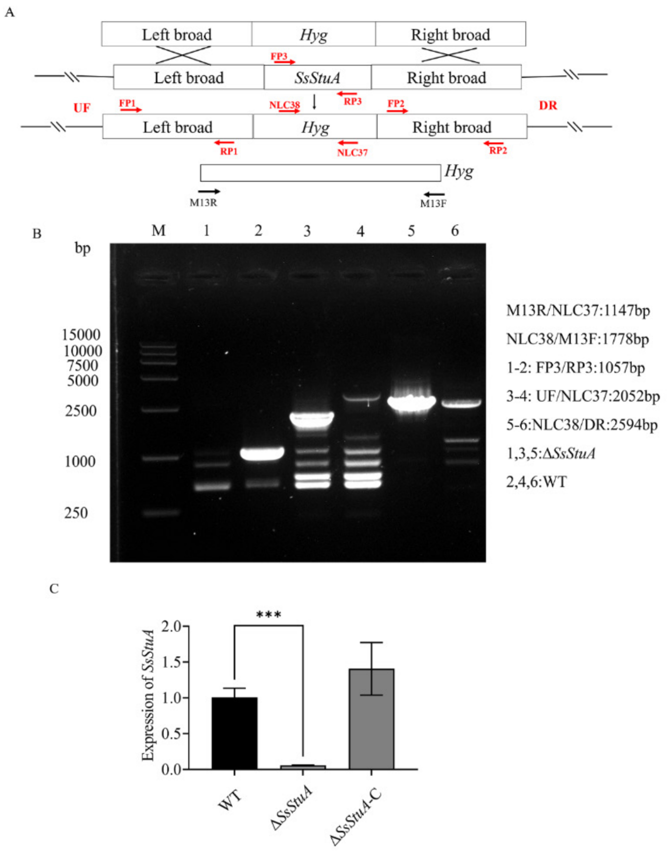

Figure S1. Identification of mutants by PCR and qPCR. (A) The knockout strategy of *SsStuA* gene. (B) Knockout of *SsStuA* was verified by PCR. (C) The knockout and complementation mutants of *SsStuA* were verified by PCR. Error bars represent the SDs and significant difference was performed by *t* test (***, $p < 0.001$).

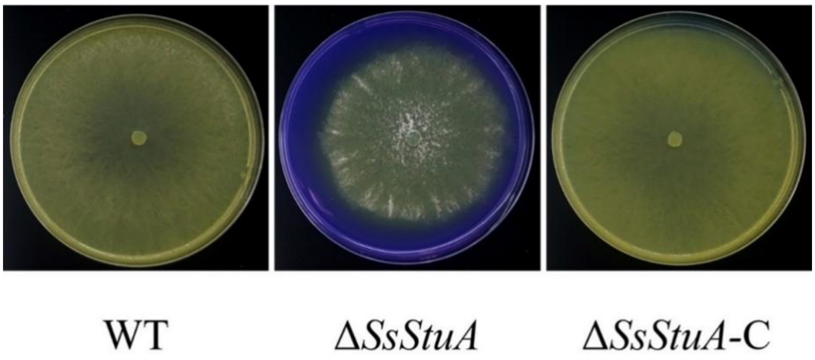

Figure S2. Δ*SsStuA* produced oxalic acid normally. Photographs were taken at 2 days post-inoculation.

Table S1. Primers used in this paper.

The name of primer	Sequence
SsStuA-F1	AAGAAGCGACAAGCACAA
SsStuA -R1	TCCTGTGTGAAATTGTTATCCGCTGGGAGGGAAACAGGTAAA
SsStuA -F2	GTCGTGACTGGGAAAACCCTGGCGAAAGCATTCTCGGCAATC
SsStuA -R2	TACCACCTCATCTCCACAAG
SsStuA -F3	TCTAAAGGGTGTTTGGTAA
SsStuA -R3	TAGTATGACTGTGGTGCTGA
M13R	AGCGGATAACAATTTCACACAGGA
NLC37	GGATGCCTCCGCTCGAAGTA
NLC38	CGTTGCAAGACCTGCCTGAA
M13F	CGCCAGGGTTTTCCCAGTCACGAC
UF	TCGCATCCCTCGAATCGAAG
DR	ATATCAAAATCATCCATCAT
SsStuA -CF1	AAGAAGCGACAAGCACAA
SsStuA -CR1	CACTGGAACAACTGGCATGTACCACCTCATCTCCACAAG
SsStuA -CF2	CAGGTACACTTGTTTAGAGGTGGTTAGGATGATGGTATGGAT
SsStuA -CR2	TTATCGGAGTCTGTGGATTG
DW69	CATGCCAGTTGTTCCAGTG
DW70	ACCTCTAAACAAGTGTACCTG
pHIS- sscle_01g011560-F	AATACGACTCACTATAGGGCCAAGGTTCTTTCATCAAATG
pHIS - sscle_01g011560-R	CGAACGCGTGAGCTCCCCGGTGTATCAACGGATTAGATGC
pHIS - sscle_04g037170-F	AATACGACTCACTATAGGGCACCAATATCTCCGGAATCAA
pHIS - sscle_04g037170-R	CGAACGCGTGAGCTCCCCGGTATTTCGGTTTTGATAGTAG
pHIS - sscle_15g107280-F	AATACGACTCACTATAGGGCAACATTTTTGAGAAGATGGG
pHIS - sscle_15g107280-R	CGAACGCGTGAGCTCCCCGGGTTGTTTGATTTCGTTTGAC
pHIS - sscle_05g044180-F	AATACGACTCACTATAGGGCTTGTTTATATCATTTTTCTT
pHIS - sscle_05g044180-R	CGAACGCGTGAGCTCCCCGGTTTGTAAAGTGTAACCGTAG
pHIS - sscle_15g104430-F	AATACGACTCACTATAGGGCGTTAAATTATTTCCTTTCGA
pHIS - sscle_15g104430-R	CGAACGCGTGAGCTCCCCGGTGCATCCATTAACTTATTTC
pHIS - sscle_05g047950-F	AATACGACTCACTATAGGGCTATAGAGAAAGAGAAGTACA
pHIS - sscle_05g047950-R	CGAACGCGTGAGCTCCCCGGAATGAATTATTGTAATGATA
pHIS - sscle_03g026200-F	AATACGACTCACTATAGGGCTTTTGACGATGTGCGTACTG
pHIS - sscle_03g026200-R	CGAACGCGTGAGCTCCCCGGGATTGCTATTATATTGCTTG
pHIS - sscle_08g064900-F	AATACGACTCACTATAGGGCGTTGACGCCAGAGTTATGGA
pHIS - sscle_08g064900-R	CGAACGCGTGAGCTCCCCGGGATTGCGGTTTACTACACTT
AD-SsStuA-F	GAATTCATGAACCACGGTCCTCAAG
AD-SsStuA-R	CTCGAGTTACCGGCGTCGTTGTGTA
ONG-SsStuA-F	GAATGGATGAACTTTACAAAATGAACCACGGTCCTCAAGA
ONG-SsStuA-R	CATCTTATCTACATACGCTACCGGCGTCGTTGTGTAATGGTT
Actin-F	GAATGTGTAAGGCCGGTTTCGC
Actin-R	CATCCCAGTTGGTGACGACACC
QSsStuA -F	AGCCTGGTCCTGCACATTAC
QSsStuA -R	TACCTTGGCCACCCATTGAC
QSsCYP51-F	AAAACGACGTGCTCAGGCTA
QSsCYP51-R	GGAACGGGGGTTCCATCTTT
QSsBIP1-F	TGTATCAACCTTAACCCCTGCC
QSsBIP1-R	TGATAAGCTCTTCGACGCGG
QSsHAC1-F	ATGCAATCAGCAATGGGCAC
QSsHAC1-R	AGGCCGTTGTACTTTCGGTT
QSsIRE1-F	GGTCGACACAGGCCTAACAA

QSsIRE1-R	ATCAACCTGCACCATCTGGG
QGNA1-F	TATGGGAACGGGCACACTTG
QGNA1-R	CGTGCCAAATTGCTGTCCTT
QGFA1-F	ACGAACAGAGAAGCGCATGA
QGFA1-R	AGAGGAAGTTTCCGACGCTG
QUAP1-F	ACAGGCAGAACGTATTCGCA
QUAP1-R	GTGTTGGCCCACTGGTCATA
QAGM1-F	GAGGGAACACCACAAGCGTA
QAGM1-R	AACAGTGACCGACCCAACAG
QCHS1-F	CAAGCCTTTGGTCGGGTACT
QCHS1-R	CTCCTGCACAAGTAGGCTCC
QCHS2-F	TCGTTCGTGCTTTCTCGTCA
QCHS2-R	AATGTTGCAGCCAAAGCGAG
QCHS3-F	CCAAGGCCTGGTACACCTTC
QCHS3-R	ATCGTCGCGTCATCTCCAAA
QBCK1-F	ACCGCAAGATCCAAGAAGGG
QBCK1-R	CGCGAAAGATCGTCTACGGA
QPKC1-F	GGTGTGCTTGGCGTTGAAAT
QPKC1-R	AGGCGATTGTTGGAGAAGCA
QMKK1-F	GCCACACCGATGGGAAGTAA
QMKK1-R	CTGGTGTTCCTTGCGGTTTG
QSMK3-F	TCGCAGAATTACTTGGCGGT
QSMK3-R	AAAGGTCGCTTGGCCATGTA
QSWI6-F	GCCCGTTTACCCGTTCTACA
QSWI6-R	ACTACGTCCTTTGACAGCCG
Qsscle_01g011560-F	TGCAATTGGCAGCAGTCCTA
Qsscle_01g011560-R	GCAGTGTTGGACCTCTCTCC
Qsscle_04g037170-F	ACCCTCAGAAGATTGCAGCC
Qsscle_04g037170-R	GGCACCAGCTTTCTCAGCTA
Qsscle_15g107280-F	TTCCCATCCTTCACCCATGC
Qsscle_15g107280-R	TGATTTGCCTCATCCTGCGT
Qsscle_05g044180-F	TGCCATTGAGCGTGGAGATT
Qsscle_05g044180-R	GCGGCGTCTGGATATGAGAA
Qsscle_08g064900-F	AATTGCTGGTGCAGATTGCG
Qsscle_08g064900-R	ATCCAACAACGGATCCCCAC
Qsscle_15g104430-F	CAAAACGTCCATCCCCTCCA
Qsscle_15g104430-R	ATCACTAGCCACAGGCACAC
Qsscle_05g047950-F	TTATCCAGATGCCCATCGCC
Qsscle_05g047950-R	CGTGCACCTTATCGGCATTG
Qsscle_03g026200-F	CCAGATACCCACCGTCATCG
Qsscle_03g026200-R	ACCAACCTCAGCCAAGTAGC
